# *tert*-Butyl *N*-{4-[*N*-(4-hy­droxy­phen­yl)carbamo­yl]benz­yl}carbamate

**DOI:** 10.1107/S1600536812036999

**Published:** 2012-09-26

**Authors:** Hai-Yang Yu, Xin Fang, Ming-Dong Huang, Jun-Dong Wang

**Affiliations:** a College of Chemistry and Chemical Engineering, Fuzhou University, Fuzhou 350108, People’s Republic of China; b Fujian Institute of Research on the Structure of Matter, State Key Laboratory of Structural Chemistry, Chinese Academy of Sciences, Fuzhou 350002, People’s Republic of China

## Abstract

In the title compound, C_19_H_22_N_2_O_4_, the dihedral angle between the aromatic rings is 67.33 (2)°. In the crystal, mol­ecules are linked through N—H⋯O and O—H⋯O hydrogen bonds, generating a two-dimensional network lying parallel to (100). As a result of the twist of the mol­ecular skeleton and the hindrance of the *tert*-butyl groups, no π–π inter­actions exist between the aromatic rings.

## Related literature

For biochemical background, see: Jiang (2009 ▶).
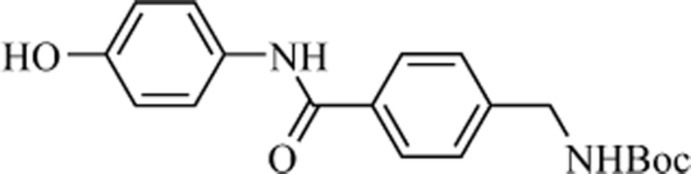


## Experimental

### 

#### Crystal data

C_19_H_22_N_2_O_4_
*M* *_r_* = 342.39
Monoclinic, 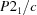

*a* = 12.289 (3) Å
*b* = 14.185 (3) Å
*c* = 10.980 (2) Å
β = 98.21 (3)°
*V* = 1894.4 (7) Å^3^
*Z* = 4
Mo *K*α radiation
μ = 0.09 mm^−1^
*T* = 293 K
0.66 × 0.66 × 0.47 mm

#### Data collection

Rigaku Saturn724 CCD diffractometer
Absorption correction: numerical (*NUMABS*; Higashi, 2000 ▶) *T*_min_ = 0.975, *T*_max_ = 0.984
15051 measured reflections
4230 independent reflections
3921 reflections with *I* > 2σ(*I*)
*R*_int_ = 0.028

#### Refinement

*R*[*F* ^2^ > 2σ(*F* ^2^)] = 0.056
*wR*(*F* ^2^) = 0.166
*S* = 1.13
4230 reflections
278 parameters
H atoms treated by a mixture of independent and constrained refinement
Δρ_max_ = 0.15 e Å^−3^
Δρ_min_ = −0.16 e Å^−3^

### 

Data collection: *CrystalClear* (Rigaku, 2007 ▶); cell refinement: *CrystalClear*; data reduction: *CrystalClear*; program(s) used to solve structure: *SHELXS97* (Sheldrick, 2008 ▶); program(s) used to refine structure: *SHELXL97* (Sheldrick, 2008 ▶); molecular graphics: *ORTEX* (McArdle, 1995 ▶); software used to prepare material for publication: *SHELXL97* and *PLATON* (Spek, 2009 ▶).

## Supplementary Material

Crystal structure: contains datablock(s) I, global. DOI: 10.1107/S1600536812036999/hb6946sup1.cif

Structure factors: contains datablock(s) I. DOI: 10.1107/S1600536812036999/hb6946Isup2.hkl

Supplementary material file. DOI: 10.1107/S1600536812036999/hb6946Isup3.cml

Additional supplementary materials:  crystallographic information; 3D view; checkCIF report

## Figures and Tables

**Table 1:** Hydrogen-bond geometry (Å, °)

*D*—H⋯*A*	*D*—H	H⋯*A*	*D*⋯*A*	*D*—H⋯*A*
N1—H1*N*1⋯O4^i^	0.88 (2)	2.10 (2)	2.949 (2)	160.6 (18)
N2—H2*N*2⋯O2^ii^	0.85 (2)	2.10 (2)	2.897 (2)	156.8 (19)
O4—H4*O*4⋯O3^iii^	0.87 (3)	1.78 (3)	2.6534 (18)	175 (3)

Symmetry codes: (i) 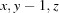; (ii) 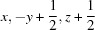; (iii) 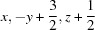.

**Fig. 1.**
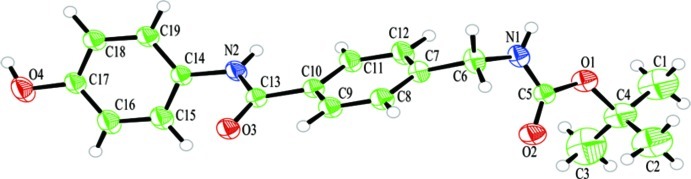
The crystal structure of (I), drawn with 30% probability displacement ellipsoids. Hydrogen atoms are shown as spheres of arbitrary radii.

**Fig. 2.**
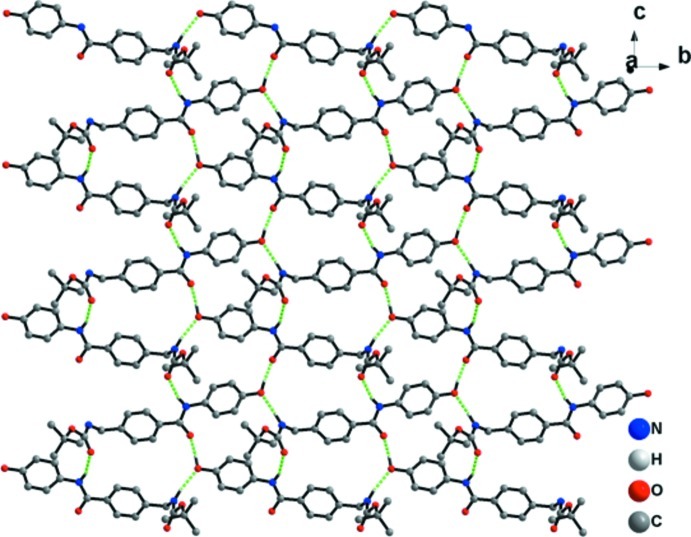
A supermolecular planar network formed by hydrogen bonds. Hydrogen atoms except those forming hydrogen bonds are omitted for clarity.

**Fig. 3.**
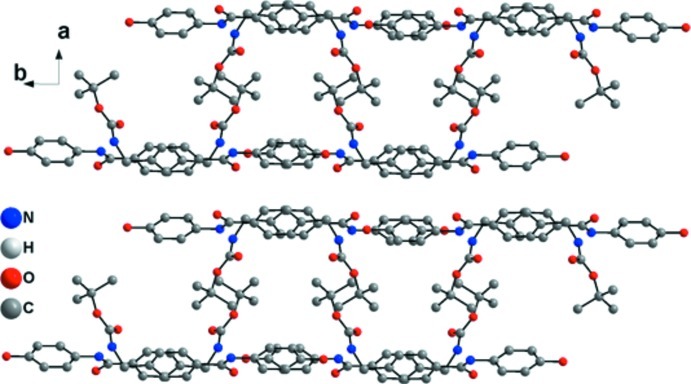
Packing of the planar networks. All hydrogen atoms are omitted for clarity.

## supplementary crystallographic information

### Comment

The title compound (I), (Fig. 1) is prepared as an intermediate of urokinase inhibitor (Jiang *et al.*, 2009). The molecule is constructed by the main body of 4-(aminomethyl)benzylcarbonic, the N-protection group of *tert*-butyl oxycarbonyl, and the 4-aminophenol amidated the 4-(aminomethyl)benzylcarbonic acid. In the crystal, dihedral angle between the planes of N-protection carbamate (C4, C5, O1, O2, N1) and methylene benzene moieties is 80.66 (8)°, and between the benzene rings linked by amide bond is 67.33 (2)°. Strong hydrogen bonds, N1—H···O4, N2—H···O2, and O4—H···O3, exist in the crystal packing, as listed in table 1. By these hydrogen bonds, a two-dimensional supermolecular network paralleled with (100) plane was formed (Fig. 2). The network planes packed with weak van de Walss interactions (Fig. 3), where all *tert*-butyl moieties are in one side of the network plane and interacted with the *tert*-butyl moieties of the neighbor plane, and although the aromatic backbones are face to face packed, there are not π-π interactions between the aromatic rings because of the twist of the skeletons.

### Experimental

A solution of 4-(aminomethyl)benzoic acid (1.51 g, 10 mmol), triethylamine (3 ml), tetrahydrofuran (THF, 20 ml) and 20 ml of water was stirred and added dropwise a solution of Di-*tert*-butyl dicarbonate ((Boc)_2_O, 3. 27 g, 15 mmol) in 20 ml of THF under room temperature. After addition the solution was stirred furtherly overnight under room temperature. Then the solution was concentrated under vacuum to about 30 ml. 100 ml of 5% NaHCO_3_ solution was added to the solution then the solution was extracted with dichloromethane (30 ml × 3). The water layer was acidified by 3 N HCl until pH ≈ 4 then white precipitate appeared. The precipitate was filtrated, washed with water, and dried to get white solid of N-Boc protected 4-(aminomethyl)benzoic acid (BAMBZA), 2.39 g (yield: 95%).

A solution of BAMBZA (684 mg, 2 mmol), 2-(1*H*-Benzotriazole-1-yl)-1,1,3,3-tetramethyluronium hexafluorophosphate (HBTU, 1138 mg, 3 mmol), *N*,*N*-diisopropylethylamine (DIEA, 310 mg, 2.4 mmol) and 4-aminophenol in 15 ml of DMF was stirred overnight at room temperature. Then the solution was treated with 5% NaHCO_3_ solution (150 mL) and the precipitate was filtrated, washed with water, dried, and purification by column chromatography (dichloromethane/methanol, 50:1) to give white solid of *tert*-butyl 4-(4-hydroxyphenylcarbamoyl)benzylcarbamate, 551 mg (yield: 80%). The solid was dissolved again in DMF, and filtrated. The solution was evaporated slowly at room temperature for a week to yield colourless blocks.

### Refinement

H atoms on the *tert*-butyl moiety were placed at idealized positions of CH_3_ group and refined as riding atoms with *U*_iso_(H) = 1.5 × *U*_eq_(C). Other H atoms were located in a difference Fourier map and refined isotropically, with C—H distances in the range of 0.95 to 1.04 Å, N—H distances of 0.86 and 0.90 Å, and O—H distances of 0.88 Å.

### Crystal data

**Table:**

C_19_H_22_N_2_O_4_	*F*(000) = 728
*M**_r_* = 342.39	*D*_x_ = 1.200 Mg m^−^^3^
Monoclinic, *P*2_1_/*c*	Mo *K*α radiation, λ = 0.71073 Å
Hall symbol: -p 2ybc	Cell parameters from 5464 reflections
*a* = 12.289 (3) Å	θ = 3.1–27.5°
*b* = 14.185 (3) Å	µ = 0.09 mm^−^^1^
*c* = 10.980 (2) Å	*T* = 293 K
β = 98.21 (3)°	Block, colourless
*V* = 1894.4 (7) Å^3^	0.66 × 0.66 × 0.47 mm
*Z* = 4	

### Data collection

**Table:**

Rigaku Saturn724 CCD diffractometer	4230 independent reflections
Radiation source: fine-focus sealed tube	3921 reflections with *I* > 2σ(*I*)
Graphite monochromator	*R*_int_ = 0.028
Detector resolution: 28.5714 pixels mm^-1^	θ_max_ = 27.5°, θ_min_ = 3.1°
ω scans	*h* = −15→15
Absorption correction: numerical (*NUMABS*; Higashi, 2000)	*k* = −18→17
*T*_min_ = 0.975, *T*_max_ = 0.984	*l* = −14→14
15051 measured reflections	

### Refinement

**Table:**

Refinement on *F*^2^	Primary atom site location: structure-invariant direct methods
Least-squares matrix: full	Secondary atom site location: difference Fourier map
*R*[*F*^2^ > 2σ(*F*^2^)] = 0.056	Hydrogen site location: inferred from neighbouring sites
*wR*(*F*^2^) = 0.166	H atoms treated by a mixture of independent and constrained refinement
*S* = 1.13	*w* = 1/[σ^2^(*F*_o_^2^) + (0.0857*P*)^2^ + 0.2768*P*] where *P* = (*F*_o_^2^ + 2*F*_c_^2^)/3
4230 reflections	(Δ/σ)_max_ < 0.001
278 parameters	Δρ_max_ = 0.15 e Å^−^^3^
0 restraints	Δρ_min_ = −0.16 e Å^−^^3^

### Special details

**Table:**

Geometry. All e.s.d.'s (except the e.s.d. in the dihedral angle between two l.s. planes) are estimated using the full covariance matrix. The cell e.s.d.'s are taken into account individually in the estimation of e.s.d.'s in distances, angles and torsion angles; correlations between e.s.d.'s in cell parameters are only used when they are defined by crystal symmetry. An approximate (isotropic) treatment of cell e.s.d.'s is used for estimating e.s.d.'s involving l.s. planes.
Refinement. Refinement of *F*^2^ against ALL reflections. The weighted *R*-factor *wR* and goodness of fit *S* are based on *F*^2^, conventional *R*-factors *R* are based on *F*, with *F* set to zero for negative *F*^2^. The threshold expression of *F*^2^ > σ(*F*^2^) is used only for calculating *R*-factors(gt) *etc*. and is not relevant to the choice of reflections for refinement. *R*-factors based on *F*^2^ are statistically about twice as large as those based on *F*, and *R*- factors based on ALL data will be even larger.

### Fractional atomic coordinates and isotropic or equivalent isotropic displacement parameters (Å^2^)

**Table:**

	*x*	*y*	*z*	*U*_iso_*/*U*_eq_	
N1	0.23491 (12)	0.02867 (9)	0.32424 (14)	0.0551 (3)	
H1N1	0.2354 (16)	−0.0024 (14)	0.394 (2)	0.066 (5)*	
N2	0.18621 (12)	0.52555 (9)	0.44084 (12)	0.0525 (3)	
H2N2	0.2117 (16)	0.4901 (14)	0.5002 (19)	0.064 (5)*	
O1	0.39656 (9)	−0.03555 (9)	0.32021 (10)	0.0611 (3)	
O2	0.31884 (11)	0.05183 (9)	0.15710 (10)	0.0663 (3)	
O3	0.11660 (10)	0.53752 (7)	0.24078 (10)	0.0561 (3)	
O4	0.18329 (14)	0.91196 (8)	0.53081 (13)	0.0774 (4)	
H4O4	0.164 (2)	0.9257 (19)	0.602 (3)	0.099 (8)*	
C1	0.5584 (2)	−0.1202 (3)	0.3697 (3)	0.1294 (12)	
H1A	0.5154	−0.1748	0.3827	0.194*	
H1B	0.5715	−0.0840	0.4443	0.194*	
H1C	0.6273	−0.1395	0.3463	0.194*	
C2	0.4655 (3)	−0.1180 (3)	0.1525 (3)	0.1287 (12)	
H2A	0.4259	−0.1732	0.1715	0.193*	
H2B	0.5310	−0.1366	0.1204	0.193*	
H2C	0.4200	−0.0809	0.0923	0.193*	
C3	0.5595 (2)	0.0279 (3)	0.2484 (4)	0.1448 (14)	
H3A	0.5187	0.0646	0.1840	0.217*	
H3B	0.6295	0.0113	0.2256	0.217*	
H3C	0.5704	0.0641	0.3230	0.217*	
C4	0.49650 (16)	−0.06027 (19)	0.2683 (2)	0.0810 (6)	
C5	0.31757 (12)	0.01781 (10)	0.25879 (13)	0.0492 (3)	
C6	0.13695 (15)	0.08113 (12)	0.2755 (2)	0.0630 (4)	
H6A	0.077 (2)	0.0590 (17)	0.321 (2)	0.094 (7)*	
H6B	0.1167 (18)	0.0648 (15)	0.184 (2)	0.077 (6)*	
C7	0.14719 (12)	0.18674 (10)	0.28959 (14)	0.0501 (3)	
C8	0.09103 (14)	0.24548 (12)	0.20160 (15)	0.0597 (4)	
H8	0.0469 (17)	0.2169 (14)	0.129 (2)	0.075 (6)*	
C9	0.09462 (14)	0.34193 (12)	0.21584 (14)	0.0568 (4)	
H9	0.0569 (19)	0.3852 (16)	0.157 (2)	0.086 (6)*	
C10	0.15430 (11)	0.38288 (10)	0.31938 (12)	0.0435 (3)	
C11	0.21137 (15)	0.32443 (11)	0.40761 (15)	0.0597 (4)	
H11	0.2557 (17)	0.3495 (15)	0.481 (2)	0.077 (6)*	
C12	0.20749 (16)	0.22745 (12)	0.39187 (17)	0.0649 (5)	
H12	0.252 (2)	0.1880 (19)	0.452 (2)	0.105 (8)*	
C13	0.15207 (11)	0.48764 (10)	0.33042 (12)	0.0435 (3)	
C14	0.18580 (13)	0.62478 (10)	0.46470 (13)	0.0482 (3)	
C15	0.23604 (16)	0.68721 (12)	0.39467 (16)	0.0615 (4)	
H15	0.2717 (16)	0.6622 (14)	0.3276 (18)	0.070 (5)*	
C16	0.23500 (18)	0.78243 (12)	0.41852 (17)	0.0667 (5)	
H16	0.2678 (19)	0.8254 (17)	0.369 (2)	0.090 (7)*	
C17	0.18335 (14)	0.81648 (11)	0.51345 (14)	0.0541 (4)	
C18	0.13337 (14)	0.75444 (11)	0.58466 (14)	0.0544 (4)	
H18	0.0951 (15)	0.7789 (13)	0.6534 (18)	0.066 (5)*	
C19	0.13457 (14)	0.65860 (11)	0.55987 (13)	0.0526 (4)	
H19	0.1014 (16)	0.6117 (14)	0.6099 (18)	0.069 (5)*	

### Atomic displacement parameters (Å^2^)

**Table:**

	*U*^11^	*U*^22^	*U*^33^	*U*^12^	*U*^13^	*U*^23^
N1	0.0620 (8)	0.0446 (7)	0.0592 (8)	0.0042 (6)	0.0101 (6)	0.0020 (6)
N2	0.0736 (9)	0.0402 (6)	0.0427 (6)	0.0055 (6)	0.0048 (6)	0.0025 (5)
O1	0.0551 (7)	0.0719 (8)	0.0553 (6)	0.0085 (5)	0.0043 (5)	0.0100 (5)
O2	0.0732 (8)	0.0750 (8)	0.0482 (6)	−0.0017 (6)	−0.0002 (5)	0.0098 (5)
O3	0.0659 (7)	0.0519 (6)	0.0486 (6)	0.0052 (5)	0.0010 (5)	0.0109 (4)
O4	0.1278 (12)	0.0449 (6)	0.0667 (8)	−0.0036 (7)	0.0390 (8)	−0.0084 (5)
C1	0.0897 (18)	0.179 (3)	0.114 (2)	0.063 (2)	−0.0057 (15)	0.018 (2)
C2	0.1022 (19)	0.180 (3)	0.1023 (19)	0.054 (2)	0.0086 (15)	−0.043 (2)
C3	0.0684 (16)	0.175 (4)	0.192 (4)	−0.0281 (18)	0.0242 (19)	0.035 (3)
C4	0.0501 (10)	0.1183 (18)	0.0739 (12)	0.0131 (10)	0.0063 (9)	0.0022 (11)
C5	0.0528 (8)	0.0459 (7)	0.0463 (7)	−0.0058 (6)	−0.0011 (6)	−0.0013 (6)
C6	0.0528 (9)	0.0475 (8)	0.0860 (12)	−0.0001 (7)	0.0008 (9)	−0.0128 (8)
C7	0.0453 (7)	0.0466 (7)	0.0575 (8)	0.0026 (6)	0.0035 (6)	−0.0084 (6)
C8	0.0637 (10)	0.0574 (9)	0.0528 (8)	−0.0003 (7)	−0.0095 (7)	−0.0097 (7)
C9	0.0647 (10)	0.0551 (9)	0.0462 (7)	0.0040 (7)	−0.0075 (7)	0.0006 (6)
C10	0.0440 (7)	0.0448 (7)	0.0414 (6)	0.0031 (5)	0.0056 (5)	−0.0008 (5)
C11	0.0732 (10)	0.0465 (8)	0.0521 (8)	0.0073 (7)	−0.0160 (7)	−0.0048 (6)
C12	0.0785 (11)	0.0458 (8)	0.0619 (9)	0.0102 (7)	−0.0190 (8)	−0.0026 (7)
C13	0.0440 (7)	0.0445 (7)	0.0425 (6)	0.0024 (5)	0.0078 (5)	0.0039 (5)
C14	0.0588 (8)	0.0416 (7)	0.0433 (7)	0.0037 (6)	0.0042 (6)	0.0011 (5)
C15	0.0806 (11)	0.0503 (8)	0.0590 (9)	0.0008 (8)	0.0284 (8)	−0.0051 (7)
C16	0.0939 (13)	0.0486 (9)	0.0643 (10)	−0.0069 (8)	0.0347 (9)	−0.0016 (7)
C17	0.0731 (10)	0.0422 (7)	0.0476 (7)	−0.0011 (7)	0.0116 (7)	−0.0038 (6)
C18	0.0690 (10)	0.0512 (8)	0.0446 (7)	0.0021 (7)	0.0139 (7)	−0.0039 (6)
C19	0.0653 (9)	0.0484 (8)	0.0451 (7)	−0.0015 (7)	0.0113 (6)	0.0025 (6)

### Geometric parameters (Å, º)

**Table:**

N1—C5	1.334 (2)	C6—C7	1.509 (2)
N1—C6	1.451 (2)	C6—H6A	1.00 (3)
N1—H1N1	0.88 (2)	C6—H6B	1.03 (2)
N2—C13	1.3380 (19)	C7—C12	1.381 (2)
N2—C14	1.4318 (18)	C7—C8	1.383 (2)
N2—H2N2	0.85 (2)	C8—C9	1.377 (2)
O1—C5	1.3353 (18)	C8—H8	0.99 (2)
O1—C4	1.468 (2)	C9—C10	1.389 (2)
O2—C5	1.2185 (18)	C9—H9	0.96 (2)
O3—C13	1.2397 (17)	C10—C11	1.387 (2)
O4—C17	1.3677 (19)	C10—C13	1.4914 (19)
O4—H4O4	0.87 (3)	C11—C12	1.387 (2)
C1—C4	1.516 (3)	C11—H11	0.97 (2)
C1—H1A	0.9600	C12—H12	0.97 (3)
C1—H1B	0.9600	C14—C15	1.376 (2)
C1—H1C	0.9600	C14—C19	1.381 (2)
C2—C4	1.515 (4)	C15—C16	1.376 (2)
C2—H2A	0.9600	C15—H15	0.98 (2)
C2—H2B	0.9600	C16—C17	1.383 (2)
C2—H2C	0.9600	C16—H16	0.95 (2)
C3—C4	1.503 (4)	C17—C18	1.379 (2)
C3—H3A	0.9600	C18—C19	1.387 (2)
C3—H3B	0.9600	C18—H18	1.006 (19)
C3—H3C	0.9600	C19—H19	0.99 (2)
			
C5—N1—C6	121.07 (16)	H6A—C6—H6B	109.2 (19)
C5—N1—H1N1	119.8 (13)	C12—C7—C8	118.19 (14)
C6—N1—H1N1	118.6 (13)	C12—C7—C6	121.70 (15)
C13—N2—C14	123.51 (12)	C8—C7—C6	120.03 (14)
C13—N2—H2N2	119.5 (13)	C9—C8—C7	120.96 (14)
C14—N2—H2N2	117.0 (13)	C9—C8—H8	120.3 (12)
C5—O1—C4	121.84 (14)	C7—C8—H8	118.7 (12)
C17—O4—H4O4	110.6 (17)	C8—C9—C10	120.87 (14)
C4—C1—H1A	109.5	C8—C9—H9	123.7 (14)
C4—C1—H1B	109.5	C10—C9—H9	115.4 (14)
H1A—C1—H1B	109.5	C11—C10—C9	118.48 (14)
C4—C1—H1C	109.5	C11—C10—C13	123.51 (12)
H1A—C1—H1C	109.5	C9—C10—C13	118.00 (12)
H1B—C1—H1C	109.5	C10—C11—C12	120.07 (14)
C4—C2—H2A	109.5	C10—C11—H11	121.7 (12)
C4—C2—H2B	109.5	C12—C11—H11	118.2 (12)
H2A—C2—H2B	109.5	C7—C12—C11	121.42 (15)
C4—C2—H2C	109.5	C7—C12—H12	119.6 (16)
H2A—C2—H2C	109.5	C11—C12—H12	118.8 (16)
H2B—C2—H2C	109.5	O3—C13—N2	121.28 (13)
C4—C3—H3A	109.5	O3—C13—C10	120.84 (12)
C4—C3—H3B	109.5	N2—C13—C10	117.85 (12)
H3A—C3—H3B	109.5	C15—C14—C19	119.27 (14)
C4—C3—H3C	109.5	C15—C14—N2	121.13 (14)
H3A—C3—H3C	109.5	C19—C14—N2	119.60 (13)
H3B—C3—H3C	109.5	C14—C15—C16	120.56 (15)
O1—C4—C3	109.5 (2)	C14—C15—H15	118.3 (12)
O1—C4—C2	109.32 (18)	C16—C15—H15	121.2 (12)
C3—C4—C2	113.6 (3)	C15—C16—C17	120.27 (16)
O1—C4—C1	102.04 (18)	C15—C16—H16	120.4 (14)
C3—C4—C1	111.0 (3)	C17—C16—H16	119.3 (14)
C2—C4—C1	110.7 (3)	O4—C17—C18	122.96 (14)
O2—C5—O1	125.72 (15)	O4—C17—C16	117.42 (14)
O2—C5—N1	123.93 (15)	C18—C17—C16	119.62 (14)
O1—C5—N1	110.35 (13)	C17—C18—C19	119.76 (14)
N1—C6—C7	114.75 (13)	C17—C18—H18	120.0 (11)
N1—C6—H6A	106.6 (14)	C19—C18—H18	120.3 (11)
C7—C6—H6A	108.5 (14)	C14—C19—C18	120.52 (14)
N1—C6—H6B	108.5 (12)	C14—C19—H19	117.1 (11)
C7—C6—H6B	109.2 (12)	C18—C19—H19	122.3 (11)

### Hydrogen-bond geometry (Å, º)

**Table:**

*D*—H···*A*	*D*—H	H···*A*	*D*···*A*	*D*—H···*A*
N1—H1*N*1···O4^i^	0.88 (2)	2.10 (2)	2.949 (2)	160.6 (18)
N2—H2*N*2···O2^ii^	0.85 (2)	2.10 (2)	2.897 (2)	156.8 (19)
O4—H4*O*4···O3^iii^	0.87 (3)	1.78 (3)	2.6534 (18)	175 (3)

Symmetry codes: (i) *x*, *y*−1, *z*; (ii) *x*, −*y*+1/2, *z*+1/2; (iii) *x*, −*y*+3/2, *z*+1/2.
